# The factors of enhancing Graduate Teaching Assistants’ Technological Pedagogical Content Knowledge (TPACK) performance in engineering curriculum teaching

**DOI:** 10.1007/s44217-022-00017-8

**Published:** 2022-11-10

**Authors:** Dedi Liu

**Affiliations:** 1 State Key Laboratory of Water Resources and Hydropower Engineering Science, Wuhan University, Wuhan, 430072 China; 2 State Experimental Teaching Centre of Water Resources and Hydropower Engineering, Wuhan University, Wuhan, 430072 China

**Keywords:** Technological Pedagogical Content Knowledge (TPACK), Graduate teaching assistants, A structural equation modeling, Engineering technology

## Abstract

Graduate teaching assistants (GTAs) play important roles in engineering education at the undergraduate level. Since there are lots of technological content knowledge (TCK) in engineering curriculums, the improvements of GTAs’ teaching skills on TCK will help the teaching effectiveness of the curriculums. As the instructor’s knowledge about technology-infused instruction for TCK is the core of the teaching skill, Technological Pedagogical Content Knowledge (TPACK) is taken as a framework to measure the extent to which instructor can teach with technology. In this study, an online questionnaires survey covering GTAs’ program coordinator, teacher, graduate student and undergraduate student has done to explore the factors of enhancing GTAs’ TPACK performance. The quantitative analyses through a structural equation modeling approach indicates that the roles of the GTAs should be clearly recognized by the teacher, program coordinators and GTAs themselves. An evaluating procedure for GTAs should be established; The attitude and self-efficacy of GTAs should be improved through the training courses and the field trips while the promising expectation from the undergraduate student on the roles of GTAs can improve the performance of GTAs’ program. Our results will be helpful not only for engineering curriculum, but also for other curriculums.

## Introduction

Engineers have been valued for their technical expertise for centuries [27]. This expertise should be provided to the next generation of engineers through the professional trainings in higher engineering-education. Thus, there are many technical curriculums in engineering major to equip students with the necessary skills for engineering success. The connection between the technological content knowledge (TCK) in a curriculum and the student’s technical and professional skills is the teaching and practice in the curriculums, which is the pedagogical knowledge of TCK. As the technologies of solving the problems referring the TCK are often switched to new versions, the instructors of the curriculum are being asked to update their skills for teaching.

In order to reduce the burden of teachers, Graduate teaching assistants (GTAs) are often adopted to lead laboratories and recitation including practice the new versions of technologies and field trip, especially at large, research-intensive universities [34, 36]. GTAs development programs (e.g., [3, 5, 9, 12, 26]) or GTAs evaluations and assessment tools (e.g., [7, 8, 30]) in engineering have been proposed to enhance the GTAs’ program performance. And the performance of GTAs’ program has been proven to be positively correlated with student learning outcomes [2, 39, 43]. The global pandemic caused by Covid-19 challenged teachers from face-to-face teaching around the world to almost instantaneously become experts in online teaching and learning [14, 33], GTAs are also not exception [1]. More than ever before, the knowledges of the technology are required for teaching activity. The knowledges of pedagogy are updated due to the changes. To develop effective teaching, especially in the changing world, the knowledges of the technology, pedagogy, and content (TPACK) are required to be completely integrated [35] to offer a conceptual bridge between the traditional approaches to instructor education. Building on the teacher knowledge research of [37, 38], the TPACK framework integrates pedagogical, content, and technological knowledges [6] to focus on meaningful connections between these three knowledge [28, 42], especially for engineering curriculum. The contents in an engineering curriculum can be classified into two parts. One is the theories and principles while the other is relating to the model constructing or practice, called the technological content knowledge (TCK). There are two types of technologies in the framework of TPACK for engineering curriculum teaching. One type is the technology knowledge as situated within the Technological Pedagogical Knowledge (TPK, e.g., digital literacy or competencies and so on) and is also part of the Pedagogical Knowledge (PK). The other is the TCK that is part of the content knowledge into the curriculum according to Shulman’s idea of Content Knowledge (CK, [37].

The main goal of GTAs’ program for engineering curriculum is how to facilitate TCK to be understood and mastered by undergraduate students through the technology with pedagogical issues. There have been increasing applications of TPACK to build instruments to assess preservice teacher’s technology integration skills [13, 16, 20, 31, 32], and several studies have also integrated the basic TPACK skills in the educational curriculum [19, 25]. However, there have been few studies that concerned on its application of TPACK in GTAs’ in teaching TCK. As there are differences and connections between the information and communication technology and TCK, there is a particular need for understanding the factors that contribute to the GTAs’ adoption of TPACK. Therefore, the main aim of this study is to explore the factors of impacting the GTAs’ TPACK performance and to improve the GTAs’ performances in the engineering curriculum teaching.

## Methods

There are many ways of measuring the TPACK, such as self-assessment surveys [35], classroom observations [22], assessment of produces or artifacts [24], and so on. In order to measure the overlapping but different facets of the situations of the GTAs’ TPACK in engineering major, a face-to-face interview and an online survey are adopted in our case study to yield an enriched, elaborated understanding the factors of impacting the performance of Graduate Teaching Assistants’ Technological Pedagogical Content Knowledge (TPACK) for Engineering curriculum teaching [10, 11, 18]. The survey took place in the context of professional curriculum in engineering majors in summer term 2021 and winter term 2021/2022. The courses of the majors contain Engineering hydrology, Hydraulics, Hydraulic and hydroelectric construction engineering, Rock mechanics, Engineering surveying, Hydraulic Structure and so on. And both TPK and TCK can be found in every course. According to the elements of the Washington Accord Graduate Attribute Profile [21], TCK are the hydrologic and hydraulic technologies including the applications of satellite technology, remote sensing, and computer technology, all of which play a role in monitoring, prediction and modeling of complex water problems and minimizing their environmental and ecological impacts. In order to improve the efficiencies of the surveying, face-to-face interview and online survey were parallelly conducted. The online survey was done by means of the QQ that is the most popular social media in China at present.

### Participants and setting

**Fig. 1:**
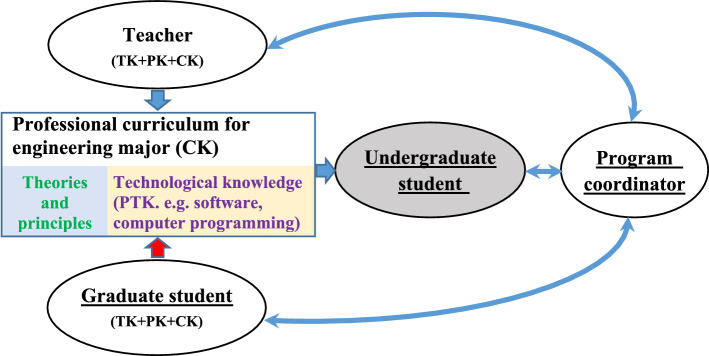
The connections between participates and the processes of teaching for undergraduate student

In the process of teaching a core professional curriculum in engineering major, there are always four types of people involving in GTAs’ program. The first one is the GTAs’ program coordinator who oversees the implementation of the program including the selection and assessment of GTAs. They connect the instructors (teacher and GTAs) and the undergraduate students and often monitor the program. The second one is the teacher who works together with GTAs. The third one is the graduate student who participates in the program. The teacher and graduate student are instructors for the curriculum, and they should have TK, PK and CK. The fourth one is the undergraduate student who is the receiver from the program. The relationships between them can be illustrated in Fig. 1. Thus, the survey recruited participants should cover all the above four types.

There have been 229 participants taken part in our survey. They are from the school of water resources and hydropower engineering, Wuhan university, China. This school is the biggest engineering school in terms of number of graduate students in the university. There are seven GTAs’ program coordinators including current and former staff. They have done the graduate affairs for many years and know the whole situations of the program and the procedure relating to teacher, graduate student and undergraduate students very well. Their experiences on the GTAs’ program are rich, showing that their answers to the survey reflect effects of the program. 47 teachers have participated in our survey. All of them have a lot of teaching experience for undergraduate students and they know their teaching curriculum very well. As only Ph.D. students have right to do teaching assistant, 93 Ph.D. graduate students (Hereafter, graduate student means Ph.D. graduate student) have taken part in the survey. Half of them have done the teaching assistant while the left will do this work in the coming semesters because the qualified teaching assistant work is one of the necessary requirements for their graduation. The purpose of the GTAs’ program is to improve the quality of engineering teaching for undergraduate students and also is to prepare the next generation of teacher. As the performer of the program, the conception and expectation of the graduate student on the program are very important for their assisting activities. Thus, their responses to our survey will be vital to find the factors that influence the program. Furthermore, there are 82 undergraduate students that have participated in the survey and all of them have already experienced in the GTAs’ program. The percentages of participants are shown in the Table 1, and the graduate students as the performers of the program occupy the biggest one.

**Table 1:** Position held by participants

Types of participants	Frequency	Percentage (%)
Program coordinator	7	3.74
Teacher (Professor, associate professor or Assistant professor)	47	25.13
Graduate student	92	49.20
Undergraduate student	41	21.93
Total	187	100

Different amounts of experience as GTAs’ program participants have been involved in the survey to increase the ability of the results discussed in this study to be transferrable to other settings. Our comprehensive account of the study also lends itself to the trustworthiness of our conclusions. There might be bias of this study due to inadequate comprehension of our stakeholder, but a lot of feedbacks from the pioneer and experienced professors can assure the accuracy of the results.

### Data collection

In order to facilitate answering the questions for the survey, online questionnaires for all kinds of participants were developed to establish basic demographic information about each participant, capture how they teach or learn the technological knowledge content, and figure out what their opinion on this program are. For every item in the survey, participants were asked to identify their level of agreement with the following choice: “strongly agree” as 5 score, “agree” as 4 score, “somewhat agree” as 3 score, “disagree” as 2 score, “strongly disagree” as 1 score.

### Data analysis

The suggestions of the program from every participant were also collected. Open-ended question was formulated in semi-structured interviews for the qualitative analysis. This survey and interview had been done during the first semester of 2020–2021. To further explore the relations between their perception, expectation, attitudes and self-esteemed to the GTAs’ TPACK program on core professional curriculum for undergraduate engineering major students, a structural equation modeling approach is chosen [23] for correcting for measurement error [4], representing the constructs as latent variables by manifest variables, specifying the relationship between latent variables and estimating the corresponding model parameters through the variance–covariance matrix. It is a useful statistical tool for exploring the entire set of relationships among latent variables [15, 17]. The acceptable (resendable) model fit of the structural model can also be evaluated by the Comparative Fit Index (CFI, ≥ 0.9) or the Tucker-Lewis Index (TLI, ≥ 0.9), or the Root Mean square error of approximation (RMSEA, ≤ 0.08) [29].

## Results and discussion

In order to explore the factors of impacting the performance of the GTAs’ TPACK program for curriculum teaching in engineering major, the quantitative results with respect to the relation between the contextual factors or latent variables (i.e. perceptions, expectation, attitudes or self-esteemed to the program) from the coordinator, teacher, graduate student and undergraduate student have been presented and discussed as well as the qualitative analysis regarding to the connections between the graduate student and the other three partners. The relationships between contextual factors or latent variables are investigated via the structural equation models. The recommendations on enhancing the performance of the program are also provided according to the quantitative and qualitative results.

**Fig. 2:**
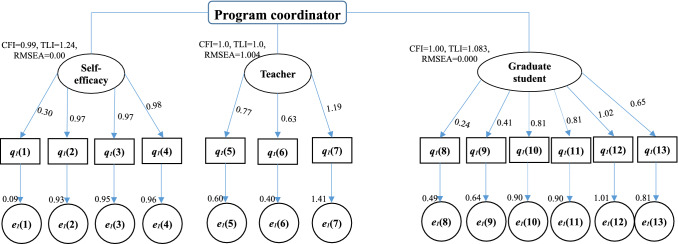
Structural equation model describing the perceptions of program coordinator on the roles played by themselves, teacher and graduate students for the GTA’s TPACK

**Fig. 3:**
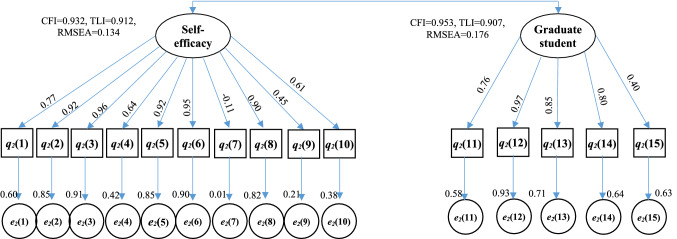
Structural equation model describing the perceptions of teacher on the roles played by themselves and graduate students for the GTA’s TPACK

**Fig. 4:**
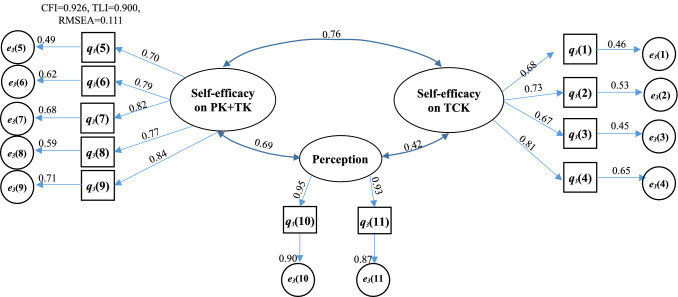
Structural equation model describing the Self-efficacy of graduate student and themselves perceptions on the GTA’s program

**Fig. 5:**
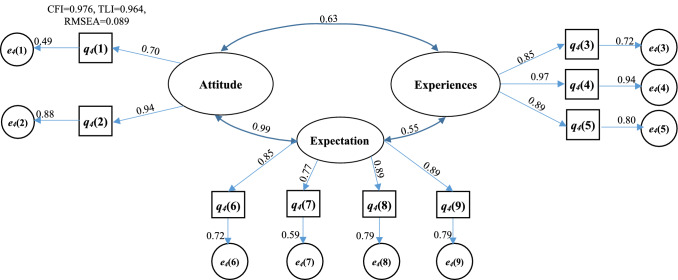
Structural equation model describing the attitude, experience and expectation of undergraduate student for GTA’s program

Cronbach’s α values were calculated to confirm the internal reliability of defined latent variables. A value of more than 0.7 shows an acceptable level of reliability of a factor or latent variable [40, 41]. The estimated values of Cronbach’s α for every latent variable were more than 0.7 shown in Appendix Tables 2, 3, 4 and 5 and that shows acceptable reliability of the collected data and extracted latent variables. The estimated values of CFI, TLI or RMSEA shown in the Figs. 2, 3,4 and 5 exhibit their good model fit according the CFI, TLI and RMSEA values.

### Analysis of the GTAs’ TPACK from the program coordinator

**Fig. 6:**
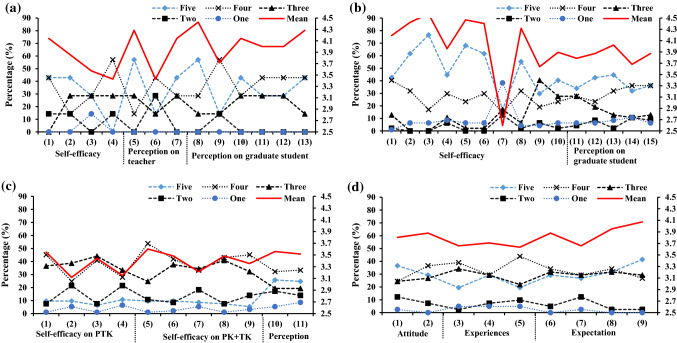
The results of surveying: **a** from the program coordinator; **b** from the teacher; **c** from the graduate students; **d** from the undergraduate students

Figure 6A shows the surveying results from the program coordinator. There are 13 questions (listed in Table 2) that are classified into three sections (Self-efficacy, perceptions on teacher and perceptions on graduate student are also taken as latent variables). The mean values of the question (4) are smaller than other 12 questions, thus it is surprise that the program coordinators have not recognized the importance of GTAs’ TPACK, which can also be found from the low mean values of the questions of their perceptions on teacher from the Fig. 6a and Fig. 2. Their perceptions on teachers’ TPACK seem to be similar as on graduate student while the current situation played roles by teacher is far away from their expectations. According to the results from the structural equation model (shown in Fig. 2), the high correlations factors in self-efficacy parcel as a latent variable are directly relating to the affairs of GTAs. Even they are not consistent with the familiar the contents of the curriculums, they know the current program very well (q_1_(2): λ = 0.97, q_1_(3): λ = 0.97 and q_1_(4): λ = 0.98), which can be found by the percentages of the Five score chosen in Fig. 6a. The high correlations factors that are q_1_(7) for the coordinator perception on teacher, while the factors q_1_(12), q_1_(10) and q_1_(11) for the perceptions on graduate student should be paid attention to improve the performance of the GTAs’ TPACK. The roles of GTAs are very positive in TCK learning for undergraduate student in coordinator perception. TK for a teacher is the dominator factor in the coordinator’s perception on teacher’s TPACK. According to the face-to-face interview for quality analysis, the procedure of selection and assessment of GTAs is not regular and should be invested more money and people to improve the GTAs’ teaching quality. There are only seven program coordinators participate our interview, but their poles plaid in this program shown in Fig. 1 are vital and their perceptions and suggestions are helpful to improve the performance of the program.

### Analysis of the GTAs’ TPACK from the teacher

Teacher plays principal role in the teaching process of a curriculum, but TCK of the curriculum is not only taught at classroom as a form of a lecture, but also should be guided to be practiced through computer software or field trip. A teacher majored in an engineering should be good at solving technical problems for teaching even for the first online teaching during the COVID-19 pandemic in 2020, thus the scores of q_2_(2) (the highest) and q_2_(7) (the lowest) are very well, which reflect the high TK levels of the teacher. There are two parcels or latent variables that are teachers’ self-efficacy on teaching and their perception on GTAs’ TPACK program as shown in Fig. 4. The high factors are their TK (q_2_(2)), CK (q_2_(3)), PK(q_2_(5) and q_2_(6)) in the self-efficacy parcel while the TK (q_2_(12)) and contribution (q_2_(13)) of GTAs are the two high factors in teacher’s perception parcel. However, the PK of GTAs is not the high factor q_2_(15), which means its importance has not been recognized by teachers. As the correlations between q_2_(4), q_2_(9), q_2_(10) and themselves esteemed on the teaching are not high (i.e., less than 0.65), the goal and the way of TCK in a curriculum are not clear by every teacher, which can directly impact their perception on GTAs. Thus, the correlations in their perception on GTAs parcel are relative lower than those in themselves self-efficacy parcel. And the percentages of the five scores in their perception are also lower.

### Analysis of the GTAs’ TPACK from the graduate student

The graduate student is the performer of GTAs program and directly determines its effectives of the program. It is interesting to find that the percentages of the four score and the three score are highest (in Fig. 6c) while the highest scores are the five and the four in Fig. 6a, b. Most of the mean values of the questions are lower than 3.7 (shown in Fig. 6c). There are 11 questions that are classified into three parcels for graduate student: the self-efficacies on TCK, the self-efficacies on PK + TK, and their perceptions on the program. The highest factors correlated to the TCK parcel are the q_3_(4) and q_3_(2). The computer programming capability as a TCK is important factor to do GTA. The updated TCKs for the curriculum are also higher correlated to the self-efficacy on TCK (shown in q_3_(4): λ = 0.81), but it has not been cared by graduate student (lower mean scores shown in Fig. 6c). Field trip can improve the self-efficacies on TCK which is higher corrected coefficient (q_3_(2): λ = 0.73), its lower mean score in Fig. 6c indicates that the field trip training should be strengthen. Beside their CK and TCK, PK might be more a challenge for graduate student to do teaching assistant program. An engineering major graduate student is often good at the TK for teaching such as the Microsoft PowerPoint software, while they are always confused the CK or TCK with the pedagogy knowledge due to no pedagogical training before. The two highest factors in the self-efficacy on PK + TK parcel are referring to the TK (q_3_(9): λ = 0.84) and TK (q_3_(7): λ = 0.82), thus training works on PK should be done for GTAs even the score of q_3_(6) referring to the PK is relatively high. The high factors for their perception on the program are the ways of selecting and evaluating for GTAs (i.e., q_3_(10): λ = 0.95 and q_3_(11): λ = 0.93) while there are high mean scores in Fig. 6(c). The relationships between the three parcels or latent variables are positive, one of them is improved and the other will be improved too. The improvement of PK + TK and TCK can lead to the improvement of their perceptions on GTAs’ program. As the path coefficient between their perception parcel and the self-efficacy is 0.69, the improvement of the perception can increase their self-efficacy on PK + TK and vice versa. Thus, the higher factors of them (i.e., ways of selecting and evaluating for GTAs, pedagogical training referring to PK and TK).

### Analysis of the GTAs’ TPACK from the undergraduate student

The effect of the program is eventually assessed by the undergraduate student. Their attitudes, experiences and expectations are composed of three parcels or latent variables. The high factor in attitude parcel is q_4_(2) in Fig. 5 (the coefficient (λ = 0.94) and its mean score (i.e., 3.88 shown in Fig. 6d) are the highest). The undergraduate student prefers making friend with GTAs to taking them only as instructors in CK and TK. The high factors in experience parcel are q_4_(4) and q_4_(5), which can further explain why the undergraduate student like to communicate with the GTAs. It has also proven that the importance of the GTAs for the teaching. The high factors in expectation parcel are q_4_(8) and q_4_(9) which are the expectations of the undergraduate student for GTAs and of their learning for the programming or software operation curriculum, especially the new requirements for GTAs during the corvid pandemic period (q_4_(9)). The scores of these two factors are also the highest in the expectation parcel (shown in Figs. 6d and 5). But it is interesting to find that the willingness of undergraduate students to being GTAs is very low if they have chance to do it from the face-to-face interview. The relationships between the three parcels are also shown in Fig. 5. The correlation coefficient between their attitudes and expectations is very close 1.0 while the coefficients between their experiences and expectations or attitudes are 0.55 and 0.63, respectively which means that much work of GTAs should be done to improve its performance.

According to the findings discussed above, all the participates complain that there is no complete rule for choosing GTAs and evaluating their performances. The importance of GTAs has still not been recognized by the program coordinator and the teacher. Some teachers even don’t know this program. If the roles of the GTAs played in a curriculum are not assigned by teacher and the GTAs themselves cannot do anything. And of course, the undergraduate student as a receiver don’t have good experiences with GTAs even they have high expectation on them. Beside the passive results of the assessment on the current situation of the program, there are also positive findings. The participating teacher or the GTAs indeed hold good TKs including the teaching tools and the professional software, even though they may lack PK while the attitudes and expectations from the undergraduate students will force the coordinator, teacher and GTAs to know the importance of the program and the advantages of the GTAs. Especially, the roles of GTAs are expected to be friends rather than only instructors, which can attract them to the curriculum or the major and can help them to relieve psychological pressure as well. Another important point from the findings is the high self-efficacies on PK, CK and TK for GTA. They can assist teaching very well, if they have been trained by the necessary pedagogical knowledge including the ways of teaching TCK.

Our findings provided insights into the development of research tools for probing GTAs performance. Objective evaluations of performances of the GTAs’ program may be an immediate indicator of success or failure of the program for the teaching in engineering curriculum. Our findings in this research may inform not only the teacher and the program coordinators to establish a system of selecting and evaluating but also the GTAs themselves with a way to know the roles and importance of GTAs.

Psychological research has addressed the influence of self-efficacy over psychometric features about teaching, but the scores of the self-efficacy of the graduate students are low, which indicates the relatively poor performance for their teaching. The self-efficacy of the teacher is good and also has led to successful teaching esteemed by themselves. And the successful mastery teaching experience is deemed as the most important source contributing to the self-efficacy in accomplishing a specific teaching activity. Furthermore, such experience can affect graduate student’s beliefs and attitudes on GTAs’ program, so the coordinator and teacher should pay more attention to the ways of acquiring mastery experience for graduate students regarding their individual characteristics. Eventually, the performances of GTAs’ program will be improved to enhance quality of the engineering education.

## Conclusion

This paper examined the GTAs’ TPACK for engineering curriculum teaching. Based on their responses through the survey, it can be concluded that more efforts should be done for improving their performances. First, their perceptions on the importance of GTAs have not been recognized by the teacher and even the coordinators as there are no rational procedures in respect of selecting and assessment of GTAs and planning program for the future. Second, the attitude and self-efficacy of GTAs should be improved through the successful teaching. The training course and field trip for their PK for the TCK might be an effective way of obtaining the success. Third, the expectation from the undergraduate student on the roles of GTAs is very promising for the implementation of the GTAs’ program. Therefore, our findings have not only found the promising future of the GTAs’ program, but also provided insights for development of GTAs’ program. As the GTAs’ program is still implementing, more researches should be done, including not only quantitative but also qualitative research, to improve curricula.

## Data Availability

All data that support the findings of this study are available from the corresponding author upon reasonable request.

## Appendix

### Interview questions

See Tables 2, 3, 4 and 5

**Table 2:** Questions for the program coordinator

Latent variable	Cronbach’s α	No	Questions
Self-efficacy	0.850	q_1_(1)	Which degree do you know the key curriculums for engineering major?
		q_1_(2)	Is there every engineering curriculum with GTAs?
		q_1_(3)	The procedure for selection and assessment of GTAs is perfect
		q_1_(4)	Do you familiar with the procedure for GTAs?
Perception on teacher	0.87	q_1_(5)	The professor who is in charge of the core curriculum can teach its CK very well
		q_1_(6)	The professor who is in charge of the core curriculum can teach its TCK
		q_1_(7)	The professor who is in charge of the core curriculum knows how to use the TK
Perception on graduate Student	0.917	q_1_(8)	The GTAs have known the CK of the curriculum very well
		q_1_(9)	The GTAs can help the students prepare the final examination
		q_1_(10)	The GTAs are better to answer the questions asked by the students, especially the new TCK
		q_1_(11)	The GTAs know the advancement of the CK and TK (e.g., software and the programming language) that can improve the learning efficiency for the underground students
		q_1_(12)	The GTAs can improve the efficiency of learning the core curriculum
		q_1_(13)	The development of the online teaching respect to the COVID-19 pandemic has pushing the improvement of teaching ways and the assessments of the performances of GTAs

**Table 3:** Questions for the teacher

Latent variable	Cronbach’s α	No	Questions
Self-efficacy	0.885	q_2_(1)	I know the PK very well
		q_2_(2)	I know how to use the TK (e.g., PowerPoint, MOOC (massive open online courses), Demo video and so on.) to aid my teaching
		q_2_(3)	I know CK of a curriculum very well, including its key points
		q_2_(4)	I know the TCK for the curriculum and know how to teach them with PK
		q_2_(5)	I can adjust the teaching plan according to the education background of the students
		q_2_(6)	There are many ways to assess the performances of students learning, including the homework, the report, the final examination, corporations among partners and so on
		q_2_(7)	I have faced TK problems when I teach online during the pandemic time
		q_2_(8)	I always update my teaching contents and tools according to the advancement of technology and research
		q_2_(9)	I always ask the student to develop a software by themselves rather than to directly apply the commercial software
		q_2_(10)	One of the purposes for the practice class is the improvement of the ability of developing software by themselves rather than the application of the commercial software
Graduate Student	0.902	q_2_(11)	The GTAs can reduce my teaching burden a lot
		q_2_(12)	I often prefer to the CK when I choose GTAs
		q_2_(13)	I often prefer to the TK when I choose GTAs
		q_2_(14)	I often train the GTAs before she or he starts to work
		q_2_(15)	GTAs not only know the advancements of the CK of a curriculum, the technological tools, and the program languages, but also know how to guide student with PK

**Table 4:** Questions for the graduate students

Latent variable	Cronbach’s α	No	Questions
Self-efficacy on TCK	0.803	q_3_(1)	I know the TCK of an engineering curriculum very well
		q_3_(2)	I can assist in the Surveying and mapping, Engineering geology curriculum s for their fieldworks including operating the instruments (e.g., Level, Robotic Total station, Perambulator)
		q_3_(3)	I know how to develop the programs for TCK of an engineering curriculum
		q_3_(4)	Besides the traditional computer languages and software applied in an engineering curriculum, I have learnt their new software and new computer languages
Self-efficacy on TK + CK	0.878	q_3_(5)	I know CK of an engineering curriculum very well before I assisting it
		q_3_(6)	I know the PK to guide students
		q_3_(7)	I can use TK including the software (e.g., flash) to help student understand the CK
		q_3_(8)	I can guide students in software operation, program development, instrument operation and so on
		q_3_(9)	I make preparation enough for answering the problems that are referring to the software operation and programming of the curriculum
Perception	0.938	q_3_(10)	I know GTAs’ work very well
		q_3_(11)	I know the ways and standards for choosing and assessing the GTAs

**Table 5:** Questions for the Undergraduate student

Latent variable	Cronbach’s α	No	Questions
Attitude	0.810	q_4_(1)	It’s necessary for me to get help from GTAs in studying an engineering curriculum
		q_4_(2)	The roles of the GTAs are not only the teaching the knowledge including theories, program developing or software operation, homework reviewing, but also are friends to us in our daily lives
Experiences	0.930	q_4_(3)	It is an effective way to consult the GTAs when I have a question to the theories (CK) of an engineering curriculum
		q_4_(4)	It is an effective way to consult the GTAs when I have a question to studying the software, and program developing of the engineering curriculum (TCK)
		q_4_(5)	GTAs often have a new idea about the curriculum, which inspires me a lot. So, I like to communicate with them
0.904 Expectation		q_4_(6)	There should be assigning GTAs in an engineering curriculum
		q_4_(7)	There should be assigning GTAs in the programming or software operation lessons for an engineering curriculum
		q_4_(8)	It will be best to arrange another lesson for software teaching, programing practice in studying the core engineering curriculum
		q_4_(9)	There will been new requirement for GTAs for online teaching due to the pandemic
